# Strengthening a culture of research dissemination: A narrative report of research day at King Faisal Hospital Rwanda, a tertiary-level teaching hospital in Rwanda

**DOI:** 10.1186/s12909-024-05736-0

**Published:** 2024-07-06

**Authors:** Kara L. Neil, Richard Nduwayezu, Belise S. Uwurukundo, Damas Dukundane, Ruth Mbabazi, Gaston Nyirigira

**Affiliations:** King Faisal Hospital Rwanda, P.O Box 2534, Kigali, Rwanda

**Keywords:** Research dissemination, Teaching hospital, Rwanda

## Abstract

**Background:**

There are significant gaps in research output and authorship in low- and middle-income countries. Research dissemination events have the potential to help bridge this gap through knowledge transfer, institutional collaboration, and stakeholder engagement. These events may also have an impact on both clinical service delivery and policy development. King Faisal Hospital Rwanda (KFH) is a tertiary-level teaching hospital located in Kigali, Rwanda. To strengthen its research dissemination, KFH conducted an inaugural Research Day (RD) to disseminate its research activities, recognize staff and student researchers at KFH, define a research agenda for the hospital, and promote a culture of research both at KFH and in Rwanda.

**Methods:**

RD was coordinated by an interdisciplinary committee of clinical and non-clinical staff at KFH. Researchers were encouraged to disseminate their research across all disciplines. Abstracts were blind reviewed using a weighted rubric and ranked by overall score. Top researchers were also awarded and recognized for their work, and equity and inclusion was at the forefront of RD programming.

**Results:**

RD had over 100 attendees from KFH and other public, private, and academic institutions. Forty-seven abstracts were submitted from the call for abstracts, with the highest proportion studying cancer (17.02%) and sexual and reproductive health (10.64%). Thirty-seven researchers submitted abstracts, and most of the principal investigators were medical doctors (35.14%), allied health professionals (27.03%), and nurses and midwives (16.22%). Furthermore, 30% of principal investigators were female, with the highest proportion of them being nurses and midwives (36.36%).

**Conclusion:**

RD is an effective way to disseminate research in a hospital setting. RD has the potential to strengthen the institution’s research agenda, engage the community in ongoing projects, and provide content-area support to researchers. Equity and inclusion should be at the forefront of research dissemination, including gender equity, authorship representation, and the inclusion of interdisciplinary health professionals. Stakeholder engagement can also be utilized to strengthen institutional research collaboration for greater impact.

## Background

Significant gaps in research output and author representation exist based on geographic region, particularly in low- and middle-income countries (LMICs). For example, one study conducted by *The Lancet Global Health* found that while 92% of articles target interventions in LMICs, only 35% of authors are actually from or work in those LMICs [1]. The *Initiative to Strengthen Health Research Capacity in Africa* identified nine key requirements for strengthening health research on the continent, including institutional support, providing research funding, promoting networks and research dissemination, and providing tools for conducting research [2]. In line with this, research dissemination events can be utilized to strengthen the research culture, institutional collaboration and knowledge transfer, and to engage stakeholders. Alongside knowledge transfer, these events can also impact both clinical service delivery and policy development [3]. This is further corroborated by an article on establishing a clinical research network in Rwanda, highlighting the importance of strengthening research partnerships and dissemination opportunities to mitigate the disease burden in Rwanda and the region [4].

King Faisal Hospital Rwanda (KFH) is a tertiary-level teaching hospital in Kigali, Rwanda. As a teaching hospital, KFH hosts hundreds of health professional students, including medical students, residents, fellows, allied health professionals, and nurses. Furthermore, KFH hosts some of Rwanda’s most highly specialized medical services and their respective subspecialty fellow trainees, including a catheterization laboratory, cardiothoracic surgery, and renal transplant surgery. While KFH previously had a focal person for education and research activities, there was no full-time team in place to manage this. Therefore, to mitigate this, KFH established a Division of Education, Training, and Research in 2021 to oversee the ongoing teaching and learning activities, including research capacity building and output. KFH also has its own Institutional Review Board (IRB) to review and approve research projects conducted at the hospital, and to monitor the overall uptake in research activity. Alongside the highly specialized services and training hosted at KFH, the hospital is putting significant effort into strengthening its research capacity and culture to ensure that evidence-based practice is at the forefront of strengthening these clinical services.

**Fig. 1:**
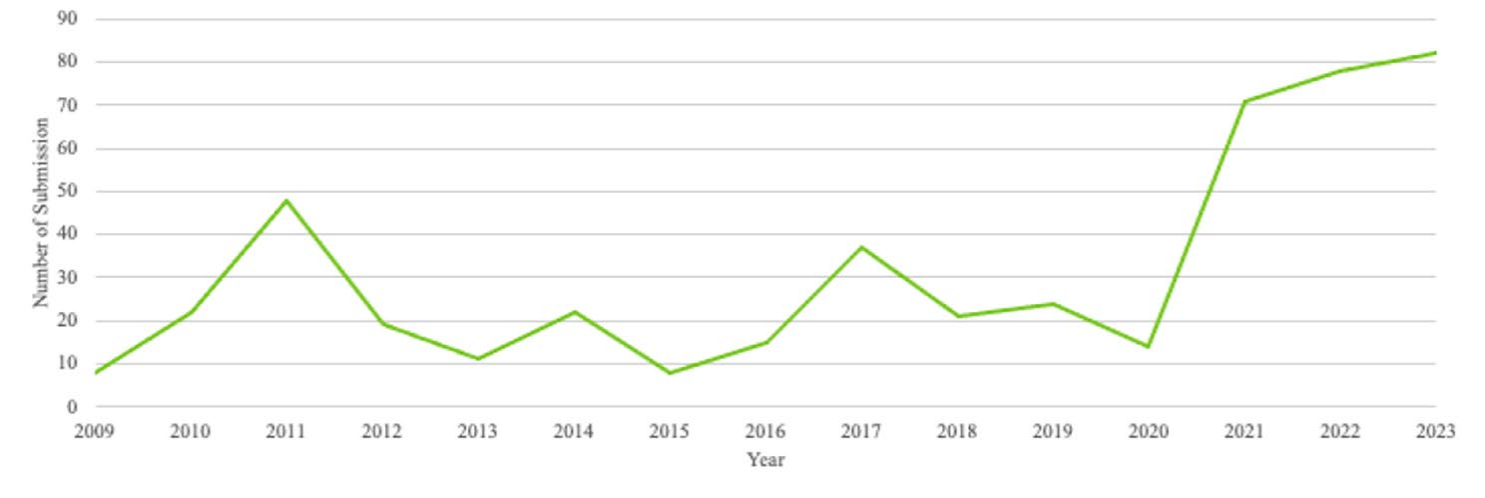
Trend of KFH IRB Submissions

The trend of research activity at KFH is also increasing, and Fig. 1 outlines the trend of KFH IRB submissions from 2009 to 2023. From 2009 to 2020, the trend in research activity was inconsistent and without a significant increase in activity. However, since 2020, there has been a significant upward trend in research activity. This is most likely attributed to the emphasis placed on evidence-based research and practice by the hospital’s leadership over the past several years. However, the numbers are still low, and further interventions are needed to improve this activity.

Research institutions and teaching hospitals are mandated to provide clinical serives, train health professionals, and conduct research. However, researchers in these institutions may not have institutionalized means of sharing their research findings with the relevant departments and leadership upon completing their research. This can result in a lack of known or implemented findings in the institutions where the research was conducted. This can also lead to the duplication of efforts, especially when research findings have not been locally disseminated or published. In response to this, having dedicated dissemination events will not only support clinical researchers to share their findings, but will also support institutions in conducting more meaningful research in relation to the institutional or national priorities, and building off of previously conducted studies.

The aim of this narrative report is to document the development and implementation of KFH’s inaugural Research Day (RD), which aimed to disseminate its research activities, recognize staff and student researchers at KFH, define a research agenda for the hospital, and promote a culture of research at KFH and more broadly in Rwanda. Furthermore, based on the output of RD, this report proposes recommendations to further strengthen research capacity and culture at KFH or through similar RD events going forward.

## Methods

RD was coordinated by an interdisciplinary clinical and non-clinical committee at KFH. Researchers were encouraged to submit and disseminate their research across all disciplines at KFH. The committee also considered ways to award and recognize researchers for their work, and ensure that the program and other logistics promoted equity and inclusion. Additionally, the committee oversaw the call for abstracts, program and participant inclusion, and the selection and awards process.

### Call for abstracts

The Directorate of Research disseminated a call for abstracts for researchers to submit their projects for poster and oral presentations. Eligible researchers included those who either work or study at KFH, or who conducted research at KFH. To encourage researchers at all stages of their study to participate, eligible abstracts included already published studies and those still in progress.

### Program and participant inclusion

To promote the inclusion of KFH staff and students in the event, the organizing committee considered the best venue for RD. As a result, RD was hosted in the KFH inpatient reception area instead of being hosted offsite, with one area for the poster display and another for the main event program. This allowed KFH staff and students to come view the poster display during their working hours without it conflicting with their regular clinical schedules. This also aimed to increase staff awareness towards the ongoing research activities at the hospital and encourage them to also get involved in research going forward.

The program for the day had several components. It commenced with a poster display, where representatives from each research team were stationed with their respective posters to answer questions and provide more information on their studies. The main program included opening remarks from the KFH Chair of the Board of Directors, a keynote speech on the importance of research dissemination from Head of Health Workforce Development at the Ministry of Health, and an overview of the state of research at KFH. The main program concluded with oral presentations and the award ceremony.

### Selection and awards

Before the event, an interdisciplinary selection committee composed of external reviewers blind-reviewed each abstract. Each abstract was evaluated using a weighted rubric, which was developed based on existing literature and the main components of an abstract. Specifically, the rubric considered 7 criteria, including clarity and organization; relevance and significance of the study; originality and innovation; methods and approach; results and findings; conclusions and implications; and grammar and writing. Within these criteria, the rubric also evaluated the overall quality of the study, adherence with ethical and legal requirements, and the validity of the findings against the methods and study design. The blind review was conducted individually by external reviewers to avoid potential biases, and reviewers were assigned to abstracts based on their expertise and the topics of the abstracts. The individual scores were then compiled, with an average taken for each abstract. The abstracts were then ranked from the highest to the lowest scores. The selection committee used these results to recommend oral and poster presenters, which included 40 posters and 7 oral presentations. In general, all abstracts meeting the minimum quality criteria were selected for poster displays. This was done to encourage researchers to disseminate their progress and increase the visibility of their work more inclusively. However, only completed studies were eligible for oral presentations.

During the event, three additional awards committees with external reviewers were established to evaluate the posters and oral presentations for one of three awards: best oral presentation, best poster presentation, and most impactful study. These committees utilized rubrics that were developed based on the main components of the abstract, along with the overall impact and presentation. The committee members reviewed the projects throughout research day, whereby the results were compiled and presented at the end of RD during the awards ceremony.

## Results

Over 100 attendees participated in the main program of RD, and additional participants attended in the poster presentation throughout the day. For the main program, attendees included key stakeholders and senior researchers from Rwanda and the region, including those with the ability to positively influence the research environment and mentor junior researchers. Specifically, participants included KFH leadership, professional councils (Rwanda Medical and Dental Council), government institutions (Ministry of Health and Rwanda Biomedical Centre), health sciences schools (University of Rwanda and University of Global Health Equity), and teaching hospitals (University Teaching Hospital of Kigali, University Teaching Hospital of Butare, and Rwanda Military Hospital), among others.

### Abstract submissions

Forty-seven abstracts were submitted from the call for abstracts, as outlined in Table 1. The highest proportion of abstracts were studying cancer (17.02%), and primarily in colorectal and breast cancer. Sexual and reproductive health was the second most represented content area, making up 10.64% of abstract submissions, followed by anesthesia and pain management (8.51%) and data science/IT (8.51%).

Table 1 Outlines the submitted abstracts by content area.

**Table 1:** Abstracts by content area

Content Area	Total	%
Cancer	8	17.02%
Other	7	14.89%
Sexual and Reproductive Health	5	10.64%
Anesthesia & Pain Management	4	8.51%
Data Science/IT	4	8.51%
Health Education	3	6.38%
Emergency & Critical Care	3	6.38%
Imaging	3	6.38%
Patient Experience	2	4.26%
Staff Experience	2	4.26%
Obstetrics and Gynecology	2	4.26%
Epilepsy	2	4.26%
Malaria	2	4.26%
**Total**	**47**	**100.00%**

### Researcher profile

Eligible researchers included KFH staff and students, as well as external researchers with projects conducted at KFH. This was decided with the aim to ensure that all disseminated research either featured KFH staff and students, or was research conducted at the hospital. Overall, 37 researchers submitted 47 abstracts. Principal Investigators (PIs) were primarily medical doctors (35.14%), allied health professionals (27.03%), and nurses and midwives (16.22%). Amongst medical doctors, anesthesia and critical care professionals represented the highest proportion of PIs (38.4%), and amongst allied health professionals, imaging services represented the highest proportion (40%). Additionally, 30% of PIs were female, with most of them being nurses or midwives (36.36%). Females comprised at least half of PIs in administration, nursing and midwifery, and data science/IT. Table 2 outlines the PIs who submitted abstracts by department and sex.

**Table 2:** Principal investigators by department & sex

	# Female	% Female	# Male	% Male	Total	% of Total
Administration	2	50%	2	50%	4	10.81%
Nursing & Midwifery	4	67%	2	33%	6	16.22%
Medical Doctors	2	15%	11	85%	13	35.14%
*Obstetrics and Gynecology*	*0*		*1*		*1*	
*Anesthesia & Critical Care*	*0*		*5*		*5*	
*Pediatrics*	*0*		*1*		*1*	
*Surgery*	*0*		*1*		*1*	
*Internal Medicine*	*1*		*2*		*3*	
*General Medicine*	*1*		*1*		*2*	
Laboratory	0	0%	2	100%	2	5.41%
Allied Health Professionals	2	20%	8	80%	10	27.03%
*Imaging Services*	*1*		*3*		*4*	
*Biomedical*	*1*		*2*		*3*	
*Physio- or Speech Therapy*	*0*		*2*		*2*	
*Non-Physician Anesthesia*	*0*		*1*		*1*	
Data Science/IT	1	50%	1	50%	2	5.41%
**Totals**	**11**	**30%**	**26**	**70%**	**37**	

### Selection process and awards

The selection committee selected seven oral presentations. Table 3 outlines the oral presentations that were selected, along with those awarded for the best oral presentation and most impactful project. Additionally, the best poster presentation was awarded to a midwife staff member who presented on strengthening family-centered maternity care at KFH.

**Table 3:** Summary of selected oral presentations

No.	Selected oral presentations
1	*Exploring the extend of resistance to ACT-treatment in malaria isolates from patients of KFHR and associated health centres in Kigali
2	*Engineering of molecular tools to predict new malaria episodes in a community
3	Barriers and facilitators to postoperative acute pain management in Rwanda from the perspective of healthcare providers: A contextualization of the theory of planned behavior.
4	Gender-based support systems influencing female students to pursue a Bachelor of Medicine, Bachelor of Surgery in Rwanda
5	Physical involvement of a woman’s male partner in childbirth process and his influence on initiation of family planning after delivery care among Rwandan couples
6	Exploring factors associated with research involvement of undergraduate students at the College of Medicine and Health Sciences, University of Rwanda
7	Mortality and sudden unexpected death in epilepsy in a cohort of 888 persons living with epilepsy in Rwanda
	*Indicates a poster prize recipient.

### Challenges

Because this was the first event of its kind at KFH, there were a few challenges in organizing and hosting the event. When the organizing committee started planning, there was a general lack of awareness on the event’s importance. Some staff questioned its benefit and why staff should be released from their clinical activities to attend. Additionally, there were few abstract submissions leading up to the submission deadline. To mitigate these issues, the committee intentionally engaged with the hospital leadership, departments, and individuals to strengthen buy in and participation in the event. This included individual meetings with department leadership to explain RD’s importance. Additionally, the RD committee membership was expanded to ensure better representation across departments and disciplines. Finally, the committee extended its submission deadline and approached researchers individually to encourage them to submit abstracts, regardless of their completion status. Because this was the first RD at KFH, engaging staff individually and at the team level helped build buy in across all levels of the institution, and ultimately increased participation in the event.

## Discussion

RD demonstrated the critical need to further strengthen research dissemination activities at KFH. The long-term aim at KFH is to promote knowledge transfer and translation through research. Research dissemination was highlighted as an initial step towards this to generate engagement and participation in the ongoing activities, and hopefully encourage junior or inactive researchers to start engaging. Specifically, RD highlighted the need to define a research agenda; promote equity and inclusion both in research activity and dissemination events; and ensure multi-institutional stakeholder collaboration in dissemination activities.

### Defining a research agenda

Common research areas were revealed through the abstract submissions, including in internal medicine (45%), obstetrics and gynecology (14%), and pediatrics (12%). However, it also revealed the need to streamline dissemination efforts through a defined hospital research agenda. This will contribute to knowledge translation in those specialties in the future, as well as more initiatives to strengthen research in those specialties. The research agenda itself may be driven by the research interests generated by the departments and researchers seen in RD. These departmental interests can then be narrowed down to specific specialties. For example, among those conducted in internal medicine, the research mainly focused on cancer, infectious diseases, and cardiovascular diseases. Integrating department or specialty-driven research priorities requires a deeper investigation into why these research areas were more frequently represented.

Additionally, many of the research projects had simple study designs, which may be attributed to limited capacity to conduct more complex projects, likely due to limited financial capacity, skills, or time. Currently, there is no policy that defines time allocation for research as a clinician. To be able to implement this research agenda and strengthen the research culture, there is a need to mobilize financial and non-financial resources that will enable the institution and researchers to conduct impactful and complex research. Ensuring equity and the distribution of research support and resources across services and departments alongside this defined research agenda is critical.

### Promoting equity and inclusion

Healthcare professionals exhibit a wide range of characteristics, including diverse social backgrounds, gender, experiences, and disability statuses [5]. As a result, healthcare institutions should adopt an inclusive research agenda that fosters cognitive diversity and encourages the sharing of innovative ideas. Such an approach ensures the development of a culturally competent workforce, ultimately reducing research biases [6]. Additionally, a culturally competent environment enhances individual motivation, leading to improved team performance [7]. This is because all healthcare providers, irrespective of their roles, contribute unique ideas and problem-solving techniques, often referred to as collective intelligence, which is essential in achieving comprehensive and unbiased research outcomes [8]. Having a diverse healthcare workforce engaged in research endeavors ensures the minimization of knowledge gaps. The multidisciplinary approach in healthcare has consistently been reflected in the highest quality of care, and it is therefore expected that it will similarly translate into the highest quality of research.

Additionally, gender equity in authorship aims to ensure equal opportunities for individuals of all genders to contribute to academic publications, which is a critical factor in professional success [9, 10]. As highlighted at KFH’s RD, individuals of all genders were welcomed and provided equal submission opportunities. This is evident in our RD researcher profile, where female PIs were 50% of administrators and 67% of nurses and midwives. Having 70% of PIs being male overall was likely influenced by the existing gender gap in medical doctors, further emphasizing the need to empower and engage women in medicine and in academic publications. Globally, the progress in women’s empowerment is reflected in the increasing number of women pursuing careers in health and academia [11]. Statistics show a significant rise in female authors in major journals, from 6% to 10% in the 1970s to 54% and 46% for first and last authorship in 2019 [12]. This progress serves as motivation for KFH, where there were gaps in female participation, highlighting the need for more intentional efforts to promote equity and inclusion in research activity and dissemination platforms.

### Stakeholder collaboration and engagement

RD revealed the importance of stakeholder collaboration to strengthen research dissemination and an overall research culture in health science institutions. As a lesson learned through RD, there is a need to streamline the way research is conducted and engage different stakeholders on this journey. To enhance and impact clinical outcomes, there is a need to strengthen research collaboration between academic institutions and hospitals. Evidence-based clinical decisions will ultimately result in higher quality healthcare by informing the development of policies and strategies. As these collective research endeavors advance, it is crucial to have a comprehensive health research policy alongside this engagement. This policy should not only serve as a guiding framework for health research within its institutions, but also ensure that the research addresses the specific needs of its communities. Students and researchers affiliated with academic institutions can contribute to fulfilling the mission of hospitals when a well-defined research agenda is in place and vice versa, and this policy will serve as the guiding principle for its implementation.

While other institutions were invited to the KFH RD, there is still a need for more intentional efforts towards institutional research collaboration and dissemination efforts. Specific ways that this can be achieved are through joint research dissemination opportunities, as well as the integration of professional societies in Rwanda, to ensure that institutions and health professions are equitably represented in these activities. Furthermore, utilizing technology can also allow for more collaboration and allow dissemination activities to be more accessible to a wider audience outside of the hospital.

### Implications for policy and practice

RD also highlighted implications for policy and practice at KFH and teaching hospitals in general. In addition to the need to define an institutional research agenda, the gaps in authorship and topic area representation across all hospital specialties suggests the need to integrate research into staff performance appraisal and promotion systems to institutionally motivate staff to participate. In doing so, the representation of all staff and respective disciplines would become more representative of the hospital itself. Furthermore, although over 100 internal and external attendees participated, and the event was hosted in the hospital for free to promote engagement, the participant number still only reflects a small proportion of the hospital, which has over 800 staff. This suggests that KFH could implement other policies or practices to motivate or require staff to participate in research-related activities. Finally, informal feedback from RD participants suggested that RD is an important step towards knowledge translation, but that additional efforts are needed alongside this event, especially towards building staff research capacity, providing resources to conduct research, and supporting those researchers with in-progress projects towards completion. Going forward, KFH will implement these recommendations towards its practices and evaluate their impact.

## Conclusion

RD provides an important platform for teaching hospitals to strengthen their research dissemination and overall research culture. RD is also an opportunity to implement the hospital’s research agenda and drive forward evidence-based practice in identified research areas. In LMICs, where there is already a significant gap in research output and authorship representation, this provides an opportunity for researchers to present and get feedback on their progress, and to motivate them to further engage in research activities. To sustain momentum and address the challenges encountered, teaching hospitals should consider RD as just one component of a broader research dissemination plan, with the wider aim of knowledge translation. By ensuring that RD is not hosted in isolation of other initiatives, this also strengthens the institutional, team-level, and individual buy in needed to strengthen RD engagement. Furthermore, when designing RD, emphasis should be given to promoting equity and inclusion in authorship, including gender, discipline, and professional experience levels. Stakeholder engagement should also be considered to strengthen institutional research collaboration for greater impact, as collaboration with other institutions can strengthen institutional research collaboration, maximizing the impact of research findings and fostering a culture of collaboration and knowledge dissemination. Going forward, KFH will continue to strengthen its research culture by leveraging RD as an initial step towards knowledge translation and implementing a defined research agenda geared towards strengthening clinical service delivery and patient outcomes.

## Data Availability

The data analyzed during this study are available from the corresponding author upon reasonable request.

## List of abbreviations

IRB: Institutional Review Board
KFH: King Faisal Hospital Rwanda
LMIC: Low- and middle-income country
PI: Principal Investigator
RD: Research Day
